# Supplementary material for: Human Beta Defensin-2 mRNA and Proteasome Subunit β Type 8 mRNA Analysis, Useful in Differentiating Skin Biopsies from Atopic Dermatitis and Psoriasis Vulgaris Patients
Source: Int J Mol Sci. 2024 Aug 24;25(17):9192. doi: 10.3390/ijms25179192 (PMC11395582; doi:10.3390/ijms25179192)
Supplement: Supplementary file 1 [file ijms-25-09192-s001.zip › ijms-3080128-supplementary.pdf]

Supplementary data:

Supplementary Data S1. Results of IVL and hBD-2 concentrations and relative expression for IVL mRNA, hBD-2 mRNA, TPP2 mRNA and PSMB8 mRNA in biopsies from patients with inflammatory skin diseases: atopic dermatitis and psoriasis vulgaris and from skin of healthy donors.

Parameter	Diagn	Cases	Median	Min	Max	LOWER QUARTILE	UPPER QUARTILE
**IVL (ng/mg)**	AD	19	12.002	0.658	189.974	7.126	29.330
**hBD-2 (pg/mg)**	AD	19	542.932	28.657	8357.794	360.472	1929.532
**IVL mRNA**	AD	19	0.287	0.127	0.673	0.212	0.410
**hBD-2 mRNA**	AD	19	0.369	0.020	1.943	0.140	0.690
**TPP2 mRNA**	AD	19	0.005	0.003	0.013	0.004	0.007
**PSMB8 mRNA**	AD	19	0.038	0.022	0.082	0.030	0.053
**IVL (ng/mg)**	PV	22	43.305	7.198	381.784	20.084	75.472
**hBD-2 (pg/mg)**	PV	22	2327.580	299.198	16708.140	1126.400	4023.572
**IVL mRNA**	PV	22	0.390	0.115	0.798	0.229	0.481
**hBD-2 mRNA**	PV	22	3.663	1.459	11.721	1.936	6.203
**TPP2 mRNA**	PV	22	0.006	0.002	0.014	0.004	0.008
**PSMB8 mRNA**	PV	22	0.036	0.018	0.170	0.029	0.044
**IVL (ng/mg)**	C	10	1.582	0.448	3.932	0.807	2.471
**hBD-2 (pg/mg)**	C	10	75.326	29.495	804.478	43.644	163.012
**IVL mRNA**	C	10	0.247	0.134	0.334	0.194	0.288
**hBD-2 mRNA**	C	10	0.002	0.0001	0.006	0.001	0.005
**TPP2 mRNA**	C	10	0.013	0.009	0.017	0.011	0.016
**PSMB8 mRNA**	C	10	0.048	0.032	0.063	0.047	0.058
**IVL (ng/mg)**	AD+PV	41	26.404	0.658	381.784	11.990	61.734
**hBD-2 (pg/mg)**	AD+PV	41	1451.948	28.657	16708.140	542.932	3508.038
**IVL mRNA**	AD+PV	41	0.333	0.115	0.798	0.215	0.476
**hBD-2 mRNA**	AD+PV	41	1.794	0.020	11.721	0.430	3.805
**TPP2 mRNA**	AD+PV	41	0.006	0.002	0.014	0.004	0.008
**PSMB8 mRNA**	AD+PV	41	0.037	0.018	0.170	0.030	0.045

AD- Atopic Dermatitis, PV- Psoriasis Vulgaris, C- controls

Supplementary Data S2. Table of counts: Itch intensity.

Value	Diagnosis	No. Cases	Percentage*
1	AD	0	0,00%
2	AD	1	5,26%
3	AD	1	5,26%
4	AD	0	0,00%
5	AD	0	0,00%
6	AD	0	0,00%
7	AD	5	26,32%
8	AD	6	31,58%
9	AD	3	15,79%
10	AD	3	15,79%
1	PV	3	13,64%
2	PV	1	4,55%
3	PV	2	9,09%
4	PV	6	27,27%
5	PV	3	13,64%
6	PV	0	0,00%
7	PV	3	13,64%
8	PV	4	18,18%
9	PV	0	0,00%
10	PV	0	0,00%

AD- Atopic Dermatitis, PV- Psoriasis Vulgaris

*Percentage of AD patients or PV patients with indicated pruritus severity.
